# Reverse Osmosis Membrane Zero Liquid Discharge for Agriculture Drainage Water Desalination: Technical, Economic, and Environmental Assessment

**DOI:** 10.3390/membranes12100923

**Published:** 2022-09-23

**Authors:** Marwa M. El Sayed, Abdelghani M. G. Abulnour, Shadia R. Tewfik, Mohamed H. Sorour, Heba A. Hani, Hayam F. Shaalan

**Affiliations:** Engineering and Pilot Plant Department, National Research Centre, Egypt El-Buhouth Street, Dokki, Cairo P.O. Box 12622, Egypt

**Keywords:** agricultural drainage water, RO membrane, desalination, evaporation, economics, ZLD

## Abstract

Agricultural drainage water (ADW) represents a potential source for fresh water after receiving appropriate treatments to satisfy the water quality requirements. Desalination of ADW with medium salinity and moderate contamination with organic and inorganic chemical pollutants could provide a techno-economically feasible approach for facing water scarcity in arid areas. The current work presents a conceptual zero liquid discharge ADW desalination system proposed to treat 300,000 m^3^/d. The system is based on pretreatment to remove impurities harmful to desalination by staged reverse osmosis (RO) membrane. The brine from the last RO stage is treated via thermal vapor compression followed by evaporation in solar ponds to recover more fresh water and salts of economic value. The essential technical features of the proposed system components are formulated. The proposed system components and its technical and economic indicators are deduced using available software for water pretreatment, RO membrane, desalination, thermal desalination, and solar evaporation ponds. The system provides total distilled water recovery of about 98% viz. 294,000 m^3^/d in addition to recovered salts of 245,000 t/y. The net cost of water production amounts to USD 0.46 /m^3^. The environmental considerations of the system are addressed and advantages of applying zero liquid discharge system are elucidated.

## 1. Introduction

Water shortage has emerged as a major issue as the world’s population grows and natural water supplies get depleted. It has been forecasted that the global demand for fresh water will exceed the supply by 40% by 2030 [1,2]. It is expected that water scarcity will increase from about one third to nearly half of the global urban population in 2050 [3]. Desalination has been widely used in regions with limited water supplies to address the problem of water shortages [4]. Desalination is the extraction of freshwater from brackish or salt water. Since the oceans cover 70% of the earth’s surface, salty water desalination has the potential to offer a plentiful freshwater supply. Desalination has proved to be a viable and reliable solution to the worldwide water shortage during the last few decades. As of 2020, the global installed desalination capacities for freshwater production are about 97.2 million m^3^/day provided by 20,971 projects [5]. The current global trend shows that membrane-based desalination technology is finding new outlets for supplying water to meet the growing water demand in most of the water-scarce countries [6,7]. The climate changes due to global warming are constantly increasing the salinity level of both land and seawater, thus reducing the availability of existing fresh water for households, agriculture, and industry. Irrigation with desalinated seawater has led to a significant increase in salinity and boron in the soils, which could affect the yield of moderately tolerant crops [8]. This has made it urgent to invent an appropriate water treatment technology that not only removes macro, micro, and nano-pollutants, but also desalinates water to a significant extent. Continued research and development of new treatment technologies are essential to improve the availability and quality of water supplies for agricultural use [9,10]. Consequently, thermal and membrane-based desalination technologies are playing an important role in solving global water scarcity problems.

Desalinated water has several advantages including (a) reduction of the possibilities of soil salinity, which has adverse effect on soil properties, (b) increasing the cultivated land area and the number of crops, and (c) improving crop quality and productivity [11]. Brackish water reverse osmosis (RO) and seawater reverse osmosis (SWRO) are the two most common desalination methods used to produce water for agriculture (SWRO). When compared to thermal desalination methods, SWRO has emerged as the most sophisticated and leading technique because of higher product water quality, reduced energy needs, and hence cheaper water costs [12].

Therefore, the aim of this work is to develop a cost effective and integrated scheme for ADW desalination with zero liquid discharge. The system is based on pretreatment to remove impurities harmful to desalination by staged reverse osmosis (RO) membrane.

## 2. Methods

### 2.1. Identification of Typical Characteristics of ADW

Agricultural drainage water (ADW) is characterized by variation of its composition from one site to another due to different effluent sources discharged to a specific drain [13]. Large drains or more specifically final drain streams exhibit moderate variation in quality as compared to small drains receiving frequent shock loads. Examples of large drains in Egypt are Bahr El-Bakar, El-Omoum, and Bahr Hadous. In this study, a typical main drain water source is considered, based on relatively stable water composition, allowing to propose an appropriate technique for treatment. Also, main drains allow considering large capacity and provide flexibility in the treatment plant site selection based on economic and environmental considerations.

Furthermore, a typical ADW treatment composition is considered. Table 1 presents the typical composition of ADW, which is mainly characterized by the following features: medium salinity in the range of brackish water, high hardness content, high suspended solids, low organic contamination, and low Fe and Mn content.

### 2.2. Rationale of Developed Proposed Treatment Scheme

The proposed treatment system is based on technically solid concepts to be applied on large scale and is environmentally accepted. The developed system comprises the following sections:

Pretreatment includes a chlorination section followed by flocculation, filtration, and sludge dewatering. This process sequence ensures the removal of microbiological contamination (e.g., algae), and performs as a softener to remove most of the Ca and Mg content, Fe and Mn, suspended solids, and organic matters. Separated sludge is dewatered and dried to be sold as a recovered by-product, while filtrate from dewatering is recycled to the process. A dual media filtration stage ensures removal of residual turbidity.

Desalination is then proposed to produce high quality water for different applications. Reverse osmosis is the most economic technique applied for that purpose. Desalination is performed by two-stage reverse osmosis (RO) to achieve maximum water recovery. The previous pretreatment allows high water recovery from RO due to eliminating most of the impurities affecting RO performance.

The concentrate from RO is further desalted to increase freshwater recovery via thermal desalination technique to produce fresh water and concentrated brine.

The concentrated brine is directed to solar evaporation ponds to recover salts.

Recovered salts from solar ponds as well as dried residues from water pretreatment present an additional economic value.

## 3. Results

### 3.1. Basic Engineering of Proposed System

Basic engineering essentially comprises material balance and technical features as well as specifications for a specified system capacity. The basis of design of the system components is presented in Table 2.

The specifications of the main streams characteristics and components specification have been identified with the aid of software available for chemical pretreatment using the following software:

- WatPro, version 4.0, Hydromantis, water treatment simulator for predicting water quality, Hamilton, Ontario, Canada
- RO using LewaPlus^®^, version 5.0, Calculation and Design Software, LANXESS, Deutschland GmbH Liquid Purification Technologies Kennedy platz 150569 Cologne, Germany,
- Thermal desalination using WT Cost II© software developed by the Bureau of Reclamation and Moch Associates, U.S. Department of the Interior, Bureau of Reclamation Technical Service Center, Denver, Colorado, according to the design features provided, and solar evaporation ponds using Excel.

### 3.2. Material Balance

**Figure 1:**
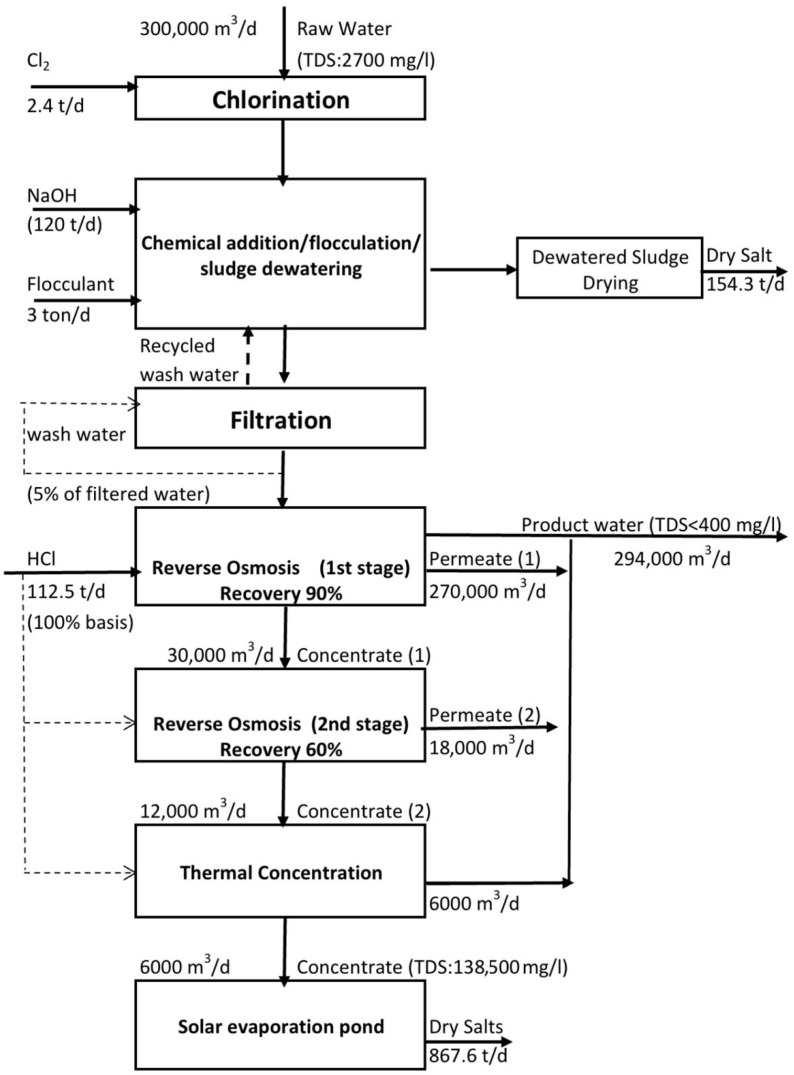
Process flow diagram, main components, and material balance of the proposed zero liquid discharge ZLD desalination system.

The material balance of the main components of the integrated ZLD desalination system is illustrated in Figure 1. Table 3 and Table 4 present the water composition of the main pretreatment streams and RO stages, respectively.

### 3.3. Technical Features and Specifications of the Proposed System

Table 5 presents the technical features and main technical specifications of the pretreatment stage, which comprises low pressure pumping station, chlorination, chemical treatment, flocculation and sedimentation, sludge dewatering, and clarified water dual media filtration.

The main technical features and specifications of two-stage RO are illustrated in Table 6**.** The first RO stage (RO1) is responsible for the recovery of fresh water up to 90% of feed water. The concentrate from this stage is directed to a second stage (RO2) to recover more fresh water of up to 60% of the quantity of the concentrate of RO1.

The concentrate of the second RO stage, (RO2) is treated in a thermal concentration unit based on thermo vapor compression (TVC) to recover up to 50% of the concentrate of RO2. The main system components are: boilers with accessories, distiller components, vessels and heat transfer tubes, pumps, degassing and chemical design units, piping including fittings and valves, electrical instrumentation and control, distiller’s support including platforms and other necessary components, thermal ejectors, chemical cleaning system, and buildings.

The concentrate from thermal concentration is directed to solar evaporation ponds in a nearby environmentally, economically, and socially accepted site to evaporate water and recover salts of economic value. The effective area of ponds amounts to 401,630 m^2^. The actual area includes banks and walkways, which amount to 50 m^2^. The walls and bottom are lined with layers of clay, geotextile, and HDPE sheets to mitigate any salty water leakage to ground water.

## 4. Financial Indicators for ZLD System

### 4.1. Cost Estimation and Financial Indicators

Cost estimates have been performed based on updated published data to the year 2021 using Chemical Engineering Cost index and the prementioned software in Section 3.2

### 4.2. Construction Cost Estimates

The summary of the construction cost of the proposed system is presented in Table 7. The construction cost is estimated to be about USD 116.4 million. Reverse osmosis units’ costs represent about 51.4% of the total costs. The total desalination cost (including RO and TVC) amounts to USD 74.92 million, representing about 64% of the total construction cost. The estimated construction costs per m^3^/day amounts to USD 396/m^3^d. These findings agree well with published work where the capital cost for brackish water RO desalination was USD 100 million at 300 m^3^/d by adopting a developed empirical correlation from hundreds of desalination plants [14].

### 4.3. Operating Costs Estimates

Table 7 presents a summary of the operating costs estimates for the proposed system. The annual operating cost is estimated to be about USD 48 million. The major cost items are chemicals and electricity, representing 53% and 21.5%, respectively.

### 4.4. Annual Production Costs Estimates

The annual production costs are the results of the annual operating cost in addition to annual capital amortization.

Table 8 and Table 9 present the estimated operating costs, revenues, and total production costs.

The net annual production costs are estimated to be about USD 56 million. Considering the revenues from recovered salts, the net annual production costs are estimated to be about USD 43.7 million. The net production cost per cubic meter of produced water is USD 0.453/m^3^.

The published range for the unit cost for brackish water RO desalination for 10,000 to 70,000 m^3^/d capacities and 2000–6000 mg/L are 0.39–0.66 USD/m^3^ [15,16]. It is worth mentioning that using electrodialysis for brackish water desalination may be lower by 5–33% according to the capacity (Generous et al., 2021). In general, the RO desalination unit cost for seawater and brackish water for capacities up to 450,000 m^3^/d is considered to be in the range of 0.7–1.4 USD/m^3^ and 0.3–0.7 USD/m^3^, respectively [14]. Moreover, Panagopoulos (2021) studied brackish water and seawater ZLD systems. He concluded that for the seawater system, the estimated cost is 0.84 USD/m^3^ which is 1.24 times higher than the brackish [17].

## 5. Environmental Considerations

The following environmental issues are addressed.

The zero-discharge desalination system provides many advantages related to environment. These include maximization of natural resources recovery and prevention of ground water contamination.

The site of the plant should be carefully selected on the bank of a main drain away from discharge points from polluted sources to avoid shock loads that sharply affect the quality of the water fed to the plant, which may cause severe deterioration of plant performance.

The site of the evaporation ponds should also be properly selected. It should be far from the residential and agricultural areas. It is preferably in a desert area near the plant site. The nature of the site and its hydrogeological characteristics should allow for environmental and economic ponds construction.

Sludge resulting from pretreatment containing mainly calcium and magnesium salts could be safely disposed of or reused after natural sun drying.

The costs of the proposed system should be re-evaluated based on the actual site conditions, which affect production cost, especially chemical water treatment, which represents a considerable proportion of the cost.

## 6. Conclusions

Agricultural drainage water (ADW) with medium salinity and heavy metal contamination could be treated to produce water of high quality for reuse. Zero liquid discharge desalination is technically and environmentally an accepted approach, by attaining maximum water and salt recovery for economic applications.

This case under study for treating ADW with a capacity of 300,000 m^3^/d concluded a total water recovery of 98% of the raw ADW. The 2% concentrated brine treated in solar evaporation ponds could recover about 245,000 ton/per year of salts, containing mainly sodium, potassium, calcium, and magnesium salts.

The estimated construction cost of the plant amounts to about USD 116.4 million, and the cost of produced water amounts to about USD 0.46/m^3^.

The cost of water production could considerably increase if lower recovery values are considered. The brine disposal, as an alternative, although may not be accepted from the environmental point of view in many situations, yet, if accepted, could be applied in specific situations with potential cost reduction.

## Figures and Tables

**Table 1:** Raw water composition.

Item	Na	K	Mg	Ca	Cl	HCO_3_	SO_4_	NO_3_
**Conc.** **(mg/L)**	596	30	140	110	1070	370	355	40
**Item**	**Fe**	**Mn**	**TDS**	**TP**	**pH**	**TSS**	**COD**	**BOD**
**Conc.** **(mg/L)**	2	1	2705	1	7.7	80	20	10

**Table 2:** Basis of design of the system components.

1. Pre-Treatment		
Raw Water Feed Flowrate		300,000 m^3^/d
Chlorine dose		8 mg/L
Sodium hydroxide dose		400 mg/L
Flocculant		10 mg/L
System		Low pressure-pumping station, In-line chlorination, chemicals (caustic soda and flocculant) addition, flocculation, and clarification, in one tank, followed by dual media filtration.
Power		1525 kW
**2. RO stages**		
	First Stage RO	Second Stage RO
Feed flow rate	300,000 m^3^/d	30,000 m^3^/d
Recovery	90%	60%
**3. Thermal Concentration**		
Feed flowrate		12,000 m^3^/day
System		Thermo vapor compression (TVC)
Power		913 kW
Recovery		50%
**4. Solar Pond**		
Feed flow rate		6000 m^3^/d
Average climate conditions:		
Temperature		24 °C
Humidity		50%
Solar irradiation		412.2 W/m^2^
Wind speed		3.5 m/s
Rainfall		65 mm/y
Elevation above sea		15

**Table 3:** Water Composition of the main pretreatment streams.

	Raw Water	Sedimentation Effluent	Filtration Effluent
Na (mg/L)	596	945	945
Ca (mg/L)	110	28	28
Mg (mg/L)	140	46	46
K (mg/L)	30	30	30
Cl (mg/L)	1070	1070	1070
SO_4_ (mg/L)	355	355	355
CO_3_ (mg/L)	-	147	147
HCO_3_ (mg/L)	370	232	232
NO_3_ (mg/L)	40	40	40
Fe (mg/L)	2	-	-
Mn (mg/L)	1	-	-
TOC (mg/L)	10	1	-
TDS (mg/L)	2705	2896	2892
pH	7.7	9.8	9.8
TOC (mg/L)	10	1	-
Turbidity (NTU)	100	5	1
Solid ppt	CaCO_3_ 205.6 mg/L		
	Mg (OH)_2_ 228.6 mg/L		

**Table 4:** Composition of two-stage RO streams *.

Stream Item	Feed Raw Water	Permeate (1)	Permeate (2)	Concentrate (1)	Concentrate (2)
Na (mg/L)	945	17.5	130	9291	23,080
Ca (mg/L)	28	0.2	1.4	278.4	694
Mg (mg/L)	46	0.3	224	457.4	1140
K (mg/L)	30	0.83	6.12	292.7	722.5
Cl (mg/L)	1070	25	1863	13,622	33,772
SO_4_ (mg/L)	355	1.3	9.7	3538.3	8831
CO_3_ (mg/L)	147	0	0	0	0.003
HCO_3_(mg/L)	232	0.04	0.28	20.3	50.3
NO_3_ (mg/L)	40	5.4	37.3	350.3	819
CO_2_ (mg/L)	0.05	0	273.6	273.6	273.6
TDS (mg/L)	2892	273.6	373.3	24,850	69,108
pH	9.8	2.32	3.18	4.9	5.26

* Before post-treatment of permeate.

**Table 5:** The main technical specifications of pretreatment components.

Item	Specifications
Chlorination	Chlorine gas vacuum feeding system 2 × 100 kg/h, with 10 chlorine gas cylinders (1 t capacity), with pumps and accessories, control and chlorine detection, and elimination system included in a separate building provided with proper ventilation.
Chemical handling system	NaOH storage tanks (3000) m^3^ capacity, pumps centrifugal, CI, 20 m^3^/h, 50 m head, feed tanks 100 m^3^ capacity feed pumps CI 10 m^3^/h, 20 m. Flocculant preparation tank (30 m^3^) and pumps, (tanks 10 m^3^ with mixers), feeding pumps 1 m^3^/h, variable speed, 5 bar pressure.
Chemical treatment/flocculation/clarification tank	4 tanks each 40 m diameter, with 4 m side wall depth, with chemical addition/flocculation section inlet, discharge wires, and sludge discharge arrangements. Mixers for rapid mixing and flocculation and sludge scraper.
Sludge dewatering	The system includes sludge pumps 8 × 10 m^3^/h, head 50 m. 4 sludge continuous belt filters, each 10 m^3^/h. 4 belt conveyers for cake (1 m width, 20 m length). 4 filtrate recycling pumps 10 m^3^/h, 20 m head.
Filtration unit	Dual media pressure filters (anthracite/sand). Operating pressure 3 bar. Diameter 3.5 m. Filtration rate 30 m^3^/m^2^ h. No. 50 With automatic backwash system.

**Table 6:** Technical specification of RO units.

Item	Specifications	
	1st Stage	2nd Stage
Feed flow rate	300,000 m^3^/d	30,000 m^3^/d
Recovery	90%	60%
Feed pressure “bar”	28.7	57.0
Pump pressure “bar”	5	5
Concentrate pressure “bar”	30	30
High pressure pump Pump/motor efficiency	84%/94%	84%/94%
High pressure pumps motors power	12,622 kW	2502 kW
Pump/motor efficiency	84%/94%	84%/94%
Booster pump motors power.	626 kW	-
System components	Cartridge filters, RO trains, RO membrane elements, high pressure pumps with energy recovery. Inter-connecting piping, post treatment, membrane cleaning unit, electrical system, and instrumentation and control, auxiliary equipment and building.	
System Configuration		
Number of stages/elements	2/13,150	1/1250
Elements number per vessel	SW HR 400 34/10	SW HR 400 34/10

**Table 7:** Summary of construction cost estimates.

No	Item	Cost USD 1000
1.	Pumping station	11,394
2.	Pretreatment	
	Chlorination	2160
	Chemical treatment/flocculation clarification and sludge dewatering	6933
	Filtration	5398
	Total pretreatment	25,885
3.	Reverse osmosis units	
	RO1	56,210
	RO2	3610
	Total RO units	59,820
4.	Thermal concentration	15,100
5.	Solar evaporation	15,600
Total		116,405

**Table 8:** Estimates of annual operating costs.

No	Item		Annual Cost USD 1000/y
1.	Electricity		10,290
		Electricity consumption: 1.47 × 10^8^ kWh/y @ USD 0.07/kW/h	
2.	Fuel	75 t/d @ USD 100/t	247
3.	Chemicals	NaOH 120 t/d @ USD 300/t Cl_2_ 2.4 t/d @ USD 150/t HCl (100% basis) 112.5 t/d @ USD 300 t/d Flocculant/anti-scaling 3.6 t/d @ USD 2000/t	11,826 118.3 11,087 2365.2
			25,396.5
4.	Membrane	Membrane lifetime 3 y	
	Replacement	Membrane cost USD 400/element	1920
5.	Maintenance	3% of capital cost	3492
6.	Labor	150 persons @ USD 15,000/y	2250
	Subtotal		43,595.5
7.	Other operating cost	10% of subtotal	4359.5
	Total operating cost		47,955.0

**Table 9:** Estimated Annual Costs, Revenues and Cost/m^3.^.

Item	Basis	Annual Cost USD 1000
Operating cost	According to Table 8	47,955
Capital amortization	Plant lifetime 20 y Interest rate 4%	8566
Total annual cost		56,521
Revenues	Mixed salts (NaCl, CaCl_2_, MgSO_4_) 245,000 t/y @ USD 50/t	12,250
Net annual cost		44,271
Cost/m^3^	Plant operation factor 0.9	USD 0.46

## Data Availability

Data available in a publicly accessible repository that does not issue DOIs. Publicly available datasets were analyzed in this study. This data can be found (https://www.davuniversity.org/images/files/study-material/PLANT%20DESIGN%20AND%20ECONOMICS%20FOR%20CHEMICAL%20ENGINEERS.pdf, accessed on 13 May 2022).
